# Evaluation of clinical guideline implementations for cardiovascular diseases by German general practitioners—Results of a web-based survey

**DOI:** 10.3389/fmed.2026.1852365

**Published:** 2026-07-03

**Authors:** Markus van der Giet, Bernhard Schwaab, Astrid Mayerböck, Konrad Klein, Olaf Randerath, Oliver Weingärtner

**Affiliations:** 1 Charité Universitätsmedizin Berlin, Berlin, Germany; 2 Curschmann Klinik, GmbH & Co. KG, Timmendorfer Strand, Germany; 3 uzbonn – Gesellschaft für empirische Sozialforschung und Evaluation, Bonn, Bonn, Germany; 4 APONTIS PHARMA Deutschland GmbH & Co. KG, Medizin, Monheim, Germany; 5 Universitätsklinikum Jena, Klinik für Innere Medizin I, Jena, Germany

**Keywords:** cardiovascular diseases, clinical guidelines, field-based physicians, implementation, web-based survey

## Abstract

**Background:**

Clinical guidelines are intended to improve the quality of medical care. The aim of this survey was to record the concrete implementation of guidelines in practice and criteria for their implementation in Germany.

**Materials and methods:**

Using a web-based questionnaire developed with medical scientific societies and uzbonn, general practitioners were able to participate anonymously in the survey between July 1, 2024, and October 31, 2024. Data were statistically evaluated using descriptive methods.

**Results:**

Four hundred thirty-seven physicians participated in the survey. 74.1% frequently implement guideline recommendations, 13.3% always, 11.2% rarely, and 1.4% never. 67.3% consider the guidelines to be too extensive. 71.6% regularly inform themselves about guideline updates. Motivators include a high recommendation rate (86.4%), recommending scientific society (78.7%), and discussion in medical training events (85.6%). The highest attention is given to National Care Guidelines (Nationale VersorgungsLeitlinie, NVL) 71.2% and guidelines of national scientific societies (65.4%). Barriers include the extent (67.3%), lack of practical relevance (51.5%), time required (47.2%), differing recommendations between various guidelines (46%), and uncertainty about validity (45.9%). Supportive measures would be a summary of essential content (98%), pocket guidelines from scientific societies (84.2%), discussions in quality circles (76.4%), and brief information in German medical journals (73.2%).

**Conclusion:**

Clinical guideline recommendations are highly accepted in everyday practice, especially the German NVL. The extent, lack of practical relevance, time required, discrepancies in recommendations, and differences in validity are significant barriers. Summaries of essential recommendations, pocket guidelines, and regular publications in medical journals could help increase application of clinical guidelines in practice.

## Introduction

Clinical guidelines are systematically developed decision-making aids for physicians with the aim of improving the quality of medical care (1). They are usually developed by clinical experts, mostly representatives of scientific societies. Their development often follows a standardized procedure that provides practical recommendations based on published clinical evidence. The recommendations are to be implemented in everyday practice through knowledge transfer (1).

Taking clinical guidelines into account in everyday practice can have a significant impact on patient health. Initial studies on the implementation of treatment recommendations in secondary prevention of cardiovascular disease in German medical practices indicate that non-compliance results in higher morbidity and mortality than consistent implementation (2–9).

The aim of our study was to determine (1) how often clinical guidelines are implemented in daily practice, (2) which factors hinder implementation, and (3) which measures could increase implementation.

## Materials and methods

This study used a cross-sectional, self-administered web-based survey design. A questionnaire was developed in collaboration with German medical societies [German Hypertension League (Deutsche Hochdruckliga e.V. DHL®)], German Society for Prevention and Rehabilitation of Cardiovascular Diseases (Deutsche Gesellschaft für Prävention und Rehabilitation von Herz-Kreislauferkrankungen e.V.), German Society for the Control of Lipid Metabolic Disorders and their Consequences (Deutsche Gesellschaft zur Bekämpfung von Fettstoffwechselstörungen und ihren Folgeerkrankungen e.V.), and uzbonn using an iterative process involving an expert advisory board and a workshop, with a focus on valid operationalization of the research questions.

The questionaire consisted of closed-ended items, mostly using four-point agreement or frequency scales.
The finalized questionnaire was programmed as a web-based survey.
Responses were submitted individually and anonymously via an online form. The responses could not be assigned to participating physicians.
Designed as a self-administered web-based survey, the study allowed respondents to interrupt and resume participation at any time.
Access to the questionnaire was provided via an SSL-encrypted connection with all pages displayed as standard web forms.

Prior to the launch all surveys underwent both automatic and manual testing. By generating random test cases, the survey system allowed precise verification of the filter guidance. In addition, the questionnaires were thoroughly checked by a team of qualified testers to ensure that the programming fully corresponded to the template. A secure connection (https) was used for the online test questionnaires to adequately protect the data entered.

Participating physicians were recruited through two channels: German general practitioners were asked by pharmaceutical representatives of APONTIS PHARMA Deutschland GmbH & Co. KG to give their consent to be contacted by mail regarding medical-scientific information and/or medical-scientific surveys. A total of 12,000 who provided written consent to such contact were invited by e-mail and 1,000 via printed flyers; the invitation described the study content, emphasized independent data analysis, and disclosed funding by APONTIS PHARMA Deutschland GmbH & Co. KG.

In concise form the flyer outlined the content and questions of the survey. The text was signed by Prof. Dr. van der Giet, Chairman of the German Hypertension League (DHL®) and the German Society for Hypertension and Prevention, as a supporter of the project. Embedded in the flyer, a QR code containing an open link could be used to access the questionnaire.

No financial compensation was paid to the consulting specialist associations or the physicians participating in the survey.

The invitations were sent out via the various channels mentioned above from July 1, 2024, to October 31, 2024.

After the end of the fieldwork period, the collected data was processed by uzbonn. Plausibility checks were not necessary in this study, as the web-based programming of the survey prevented implausibilities.

All analyses were performed using IBM SPSS Statistics.

The reporting of this web-based survey followed the CROSS (Consensus-Based Checklist for Reporting of Survey Studies) checklist and, for web-specific aspects, the CHERRIES (Checklist for Reporting Results of Internet E-Surveys) checklist (see Supplementary Material).

According to national regulations for anonymous survey research among physicians, formal approval by an ethics committee and written informed consent were not required. Participation in the survey was entirely voluntary. Before starting the questionnaire, participants were informed about the purpose of the study, the anonymous and aggregated analysis of the data, and the funding source; proceeding to the first survey page was considered as providing informed consent. All current requirements in guidelines for observational/claims-based data studies in Germany, legal requirements and in particular requirements regarding data protection and the protection of individuals were followed in this study.

## Results

**Figure 1:**
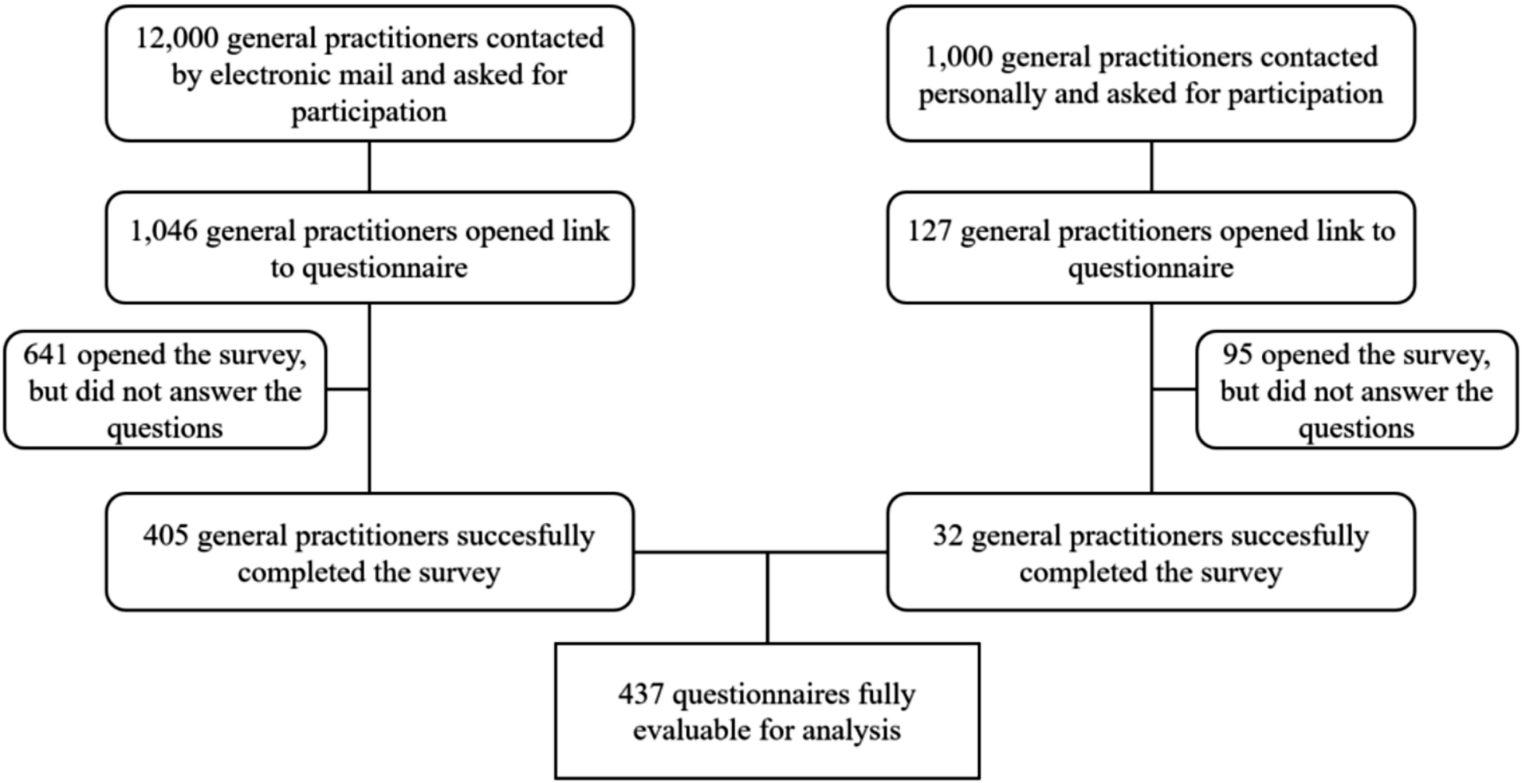
Survey CONSORT diagram, this figure provides the information, how many physicians were initially contacted, accessed the survey link, and successfully completed the questionnaire.

Of 13,000 physicians invited, 1,046 (8.0%) accessed the survey link and 437 (3.4% of all invitees; 41.8% of those who opened the link) completed the questionnaire and were included in the analysis (Figure 1).

57% of participants were general practitioners, 43% were specialists working as family doctors (14 respondents did not provide information). 45% of participants stated that they worked in a group practice (2 respondents did not provide information). 53% of participants were male. 38.7% were between 51 and 60 years old, 27.5% were older than 60, 24.7% were between 41 and 50, 8.2% were between 31 and 40, and 0.5% were 30 or younger (0.5% did not specify their age).

23% of respondents were members of the German Society for General Medicine and Family Medicine (Deutsche Gesellschaft für Allgemeinmedizin und Familienmedizin, DEGAM), 8% were members of the German Society for Cardiology (Deutsche Gesellschaft für Kardiologie, DGK), 8% were members of the German Hypertension League (DHL), 1% were members of the German Society for the Prevention of Lipid Metabolism Disorders and their Consequences (DGFF), and 1% were members of the German Society for the Prevention and Rehabilitation of Cardiovascular Diseases (DGPR). 35% were members of another professional association, and 41.4% of respondents did not belong to any professional association (multiple answers possible).

**Figure 2:**
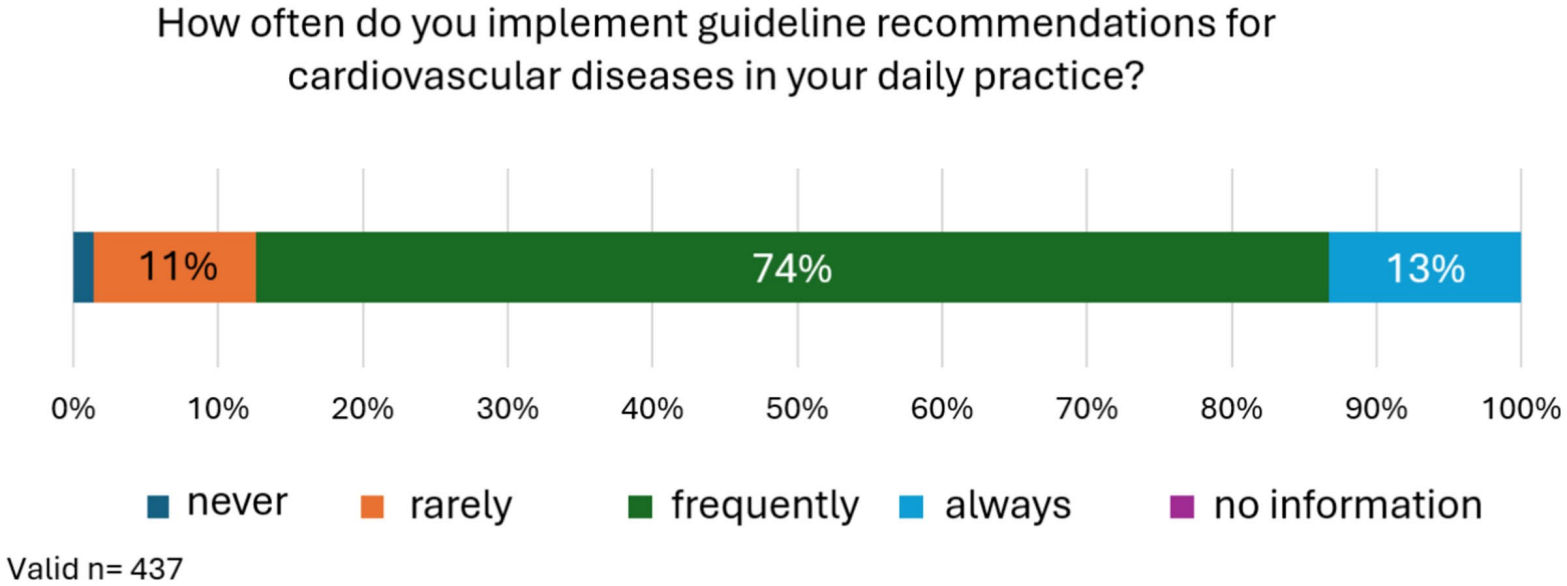
Frequency of implementation of guideline recommendations on cardiovascular diseases. Figure 2 gives the frequency of implementation of guideline recommendations on cardiovascular diseases in daily practice as given by the surveyed physicians.

Of those surveyed, 74.1% stated that they frequently implement guideline recommendations for cardiovascular diseases, 13.3% always, 11.2% rarely, and 1.4% never (Figure 2).

Among general practitioners and specialists working as family doctors, the proportion of those who frequently implement the recommendations was 74.7% (11.6% always) and 76.7% (13.2% always), respectively. General practitioners follow the guidelines frequently (53.8%) or always (38.5%). 75.4% of members of professional associations frequently implement the recommendations, 13.9% always. Among non-members, 72.2% frequently implement the recommendations and 12.8% always. The observed frequencies of implementation were comparable across age groups and between genders.

Where guidelines are not always implemented, the main obstacles were their scope (67.3%), lack of practical relevance (51.5%), time required (47.2%), conflicting recommendations between different guidelines (46%), and uncertainty about their validity (45.9%; Table 1).

**Table 1:** What prevents you from implementing guideline recommendations (*n* = 379, multiple answers possible).

Answers	Yes *n* = 379	Somewhat yes *n* = 379	Somewhat no *n* = 379	No *n* = 379	Not specified *n* = 379
Guidelines too comprehensive	*n* = 81 (21.4%)	*n* = 174 (45.9%)	*n* = 85 (22.4%)	*n* = 35 (9.2%)	*n* = 4 (1.1%)
Recommendations deviate from other guidelines	*n* = 51 (13.5%)	*n* = 123 (32.5%)	*n* = 141 (37.2%)	*n* = 62 (16.4%)	*n* = 2 (0.5%)
Updates too frequent	*n* = 39 (10.3%)	*n* = 91 (24.0%)	*n* = 163 (43.0%)	*n* = 84 (22.2%)	*n* = 2 (0.5%)
Uncertainty as to which guideline is valid	*n* = 44 (11.6%)	*n* = 130 (34.3%)	*n* = 122 (32.2%)	*n* = 81 (21.4%)	*n* = 2 (0.5%)
Takes too much time	*n* = 52 (13.7%)	*n* = 127 (33.5%)	*n* = 130 (34.3%)	*n* = 69 (18.2%)	*n* = 1 (0.3%)
Difficult or cumbersome to find	*n* = 44 (11.6%)	*n* = 110 (29.0%)	*n* = 146 (38.5%)	*n* = 77 (20.3%)	*n* = 2 (0.5%)
Guideline recommendations are too impractical	*n* = 31 (8.2%)	*n* = 164 (43.3%)	*n* = 145 (38.3%)	*n* = 37 (9.8%)	*n* = 2 (0.5%)

Differences were observed between female and male participants: 62.4% of female and 70.9% of male respondents consider the guidelines to be too extensive; 42.2% of female and 51.7% of male respondents find implementation too time-consuming; 42.2% of female and 49.2% of male respondents find differing recommendations problematic.

Furthermore, differences were also observed between individual practices and group practices. Almost half of individual practices (48.3%) agreed (yes/somewhat yes) that differing recommendations in the guidelines are an obstacle, compared to 42.1% of group practices. Uncertainty regarding the validity of guidelines was seen as an obstacle by half of the individual practices (50.7%), compared to 39.1% of respondents in group practices. 55% of respondents in individual practices and 47% of respondents in group practices felt that the recommendations were too impractical. 51.2% in solo practices and 42.8% of respondents in group practices felt that implementation was too time-consuming.

Motivators for implementing clinical guideline recommendations were the degree of recommendation (86.4%), the recommending professional association (78.7%), and discussion of the topic in continuing medical education events (85.6%; Table 2).

**Table 2:** What motivates you to implement the guideline recommendations? (*n* = 431, multiple answers possible).

Answers	Yes *n* = 431	Somewhat yes *n* = 431	Somewhat no *n* = 431	No *n* = 431	Not specified *n* = 431
Degree of recommendation in guidelines	*n* = 189 (43.9%)	*n* = 183 (42.5%)	*n* = 47 (10.9%)	*n* = 12 (2.8%)	*n* = 0 (0.0%)
Recommending professional association	*n* = 149 (34.6%)	*n* = 190 (44.1%)	*n* = 74 (17.2%)	*n* = 18 (4.2%)	*n* = 0 (0.0%)
Discussion in certified medical continuing education events	*n* = 190 (44.1%)	*n* = 179 (41.3%)	*n* = 45 (10.4%)	*n* = 16 (3.7%)	*n* = 1 (0.2%)

**Figure 3:**
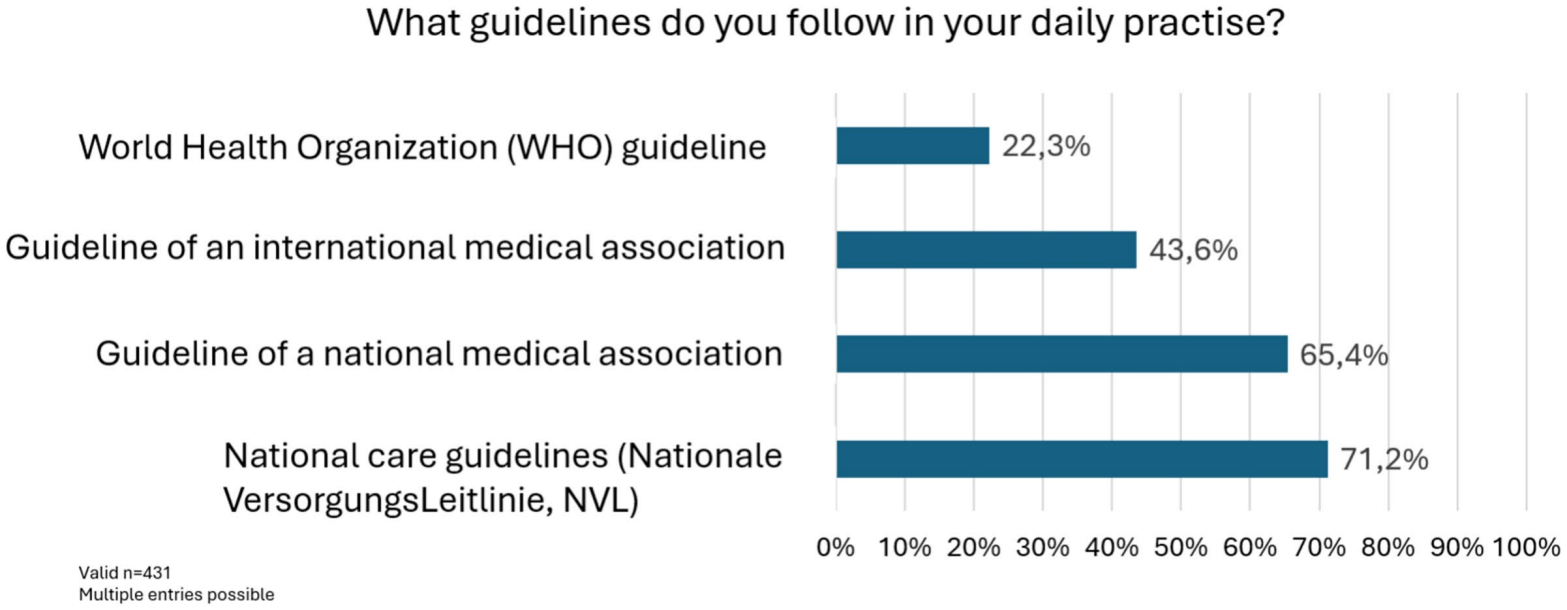
Ranking of various clinical guidelines in terms of their consideration in everyday practice. The recommendations of clinical guidelines may vary between different institutions. Figure 3 gives a ranking in terms of following clinical guidelines from various institutions by surveyed physicians.

When asked about the order of recommendation level, national care guidelines (71.2%) and guidelines from national professional associations (65.4%) were ahead of guidelines from international professional associations (43.6%) and guidelines from the World Health Organization (WHO, 22.3%; Figure 3).

Supporting measures for implementing clinical guideline recommendations included brief summaries of key content (98%), pocket guidelines from professional associations (84.2%), discussions in quality circles (76.4%), brief information in German Medical Journals such as the Deutsche Ärzteblatt (73.2%), and commentary by experts (65.7%).

When asked about their preference for a print or digital version of supporting measures, no differences were observed.

**Figure 4:**
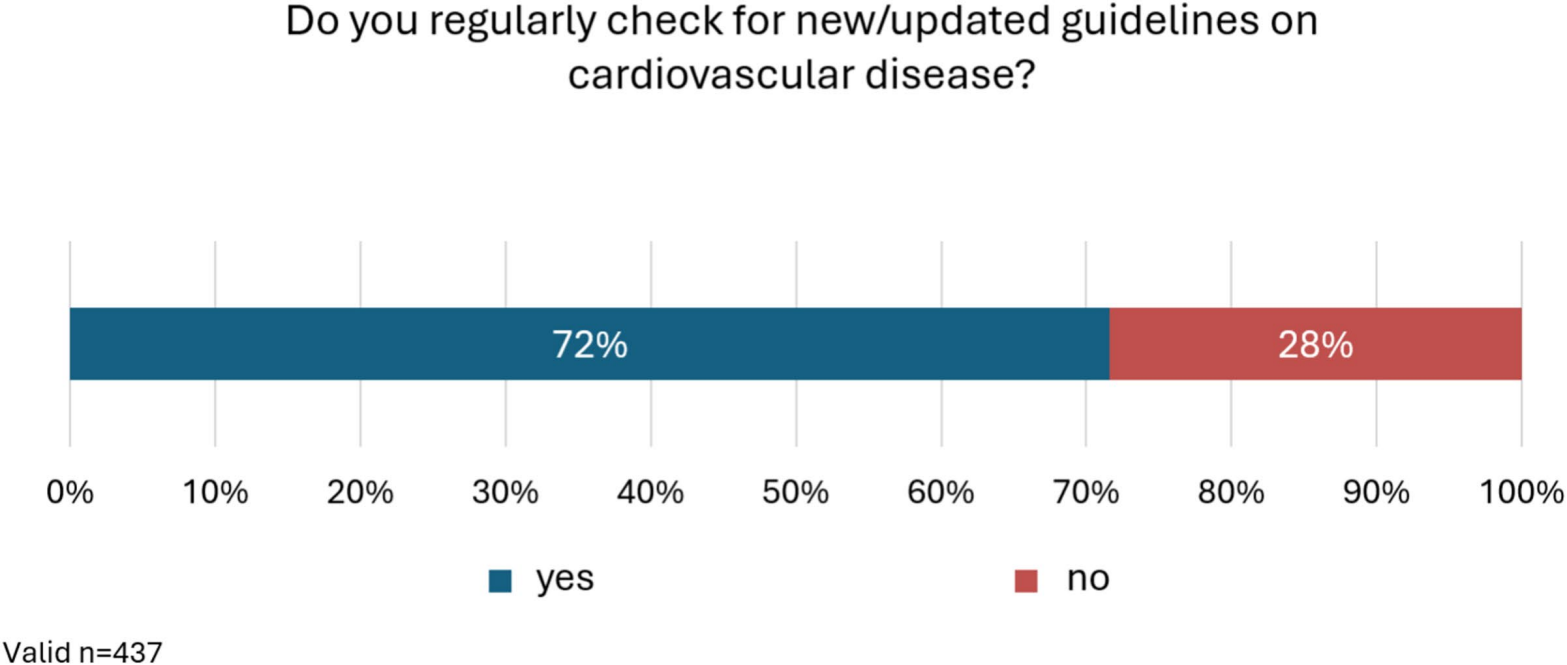
Information behavior regarding current guidelines on cardiovascular diseases. Figure 4 shows whether the respondents regularly check clinical guidelines for updates.

71.6% of respondents regularly seek information about new or current guidelines on cardiovascular diseases (76.0% of physicians in group practices, 67.1% of general practitioners, 80.4% of specialists working as family doctors, 79% of members of a professional association, and 61.1% of respondents were not members of a professional association; Figure 4).

**Figure 5:**
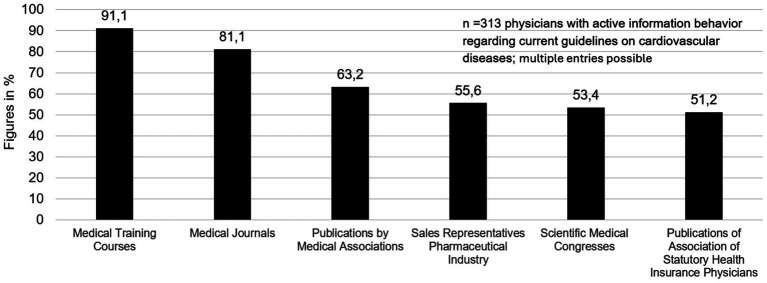
How do physicians obtain information about updates to clinical guidelines? Clinical guidelines can be published in various media. Figure 5 describes which sources the respondents use for information about clinical guidelines.

The most common sources of information on clinical guidelines are certified medical training courses (91.1%) and specialist journals (81.8%), followed by publications by medical associations (63.2%), information from pharmaceutical sales representatives (55.6%), participation in scientific conferences (53.4%), and publications by associations of statutory health insurance physicians (51.2%; Figure 5).

Compared to male participants, female participants more frequently preferred professional journals (85.9% of women vs. 78.5% of men) and certified medical training courses (94.6% of women vs. 88.3% of men) as well as information from pharmaceutical representatives (female 60.8% vs. male 51.5%).

Members of a professional association more frequently used publications from professional associations (members: 68.9% vs. non-members: 52.7%) and professional journals (members: 84.5% vs. non-members: 78.1%) as sources of information. Non-members more frequently obtain information from pharmaceutical representatives (70.9% non-members vs. 47.7% members).

## Discussion

The results of our study indicate a high overall acceptance of clinical guidelines for the treatment of cardiovascular diseases. 13.3% of respondents stated that they always implement the recommendations, and more than two-thirds implement the recommendations frequently, although we did not observe any relevant differences for the subgroups analyzed. This quantitative observation is consistent with studies on the qualitative implementation of clinical guidelines for the treatment of heart failure conducted by the Scientific Institute of the German Health Insurance AOK (Wissenschaftliches Institut der AIK, WIDO).

Nevertheless, studies on the translation of clinical guidelines into routine clinical practice have shown incomplete implementation of recommendations: some recommendations were implemented to a very high degree, whereas others were implemented only to a rather insufficient extent (10). Some possible reasons to explain this outcome can be derived from the responses of the participants in our study. One reason could be the perception that not all recommendations are clinically relevant. About half of the participants in the group we surveyed cited “lack of practical relevance” as a barrier to implementation.

Although updated clinical guideline recommendations are included in certified continuing medical education, the underlying evidence and the methodology of guideline development are rarely highlighted, meaning that the rationale for changes is not always clear to practicing physicians. This may lead to uncertainty regarding the validity of the recommendations. Therefore, the rationale and methodology behind guideline recommendations should be clearly communicated in order both to dispel scepticism about their clinical foundation and to increase confidence in the quality of the recommendations.

National guideline recommendations are apparently given greater acceptance than international guidelines. This may be due to the fact that national guidelines place a stronger focus on the requirements of the respective healthcare system, which may differ from international standards, for example with regard to cost-effectiveness and available resources.

A lower acceptance of European guidelines may also be related to the disclosure of conflicts of interest. On the one hand, the disclosure of potential conflicts of interest ensures a high degree of transparency. On the other hand, it cannot be ruled out that even declared conflicts may nonetheless influence recommendations.

In a systematic review, Tabatabavakili and colleagues reported that 45% of clinical guideline authors reported a financial conflict of interest (11). Guyatt and colleagues have stated that not only financial interests but also intellectual conflicts of interest have raised concern regarding the objectivity of guideline recommendations (12). Hinton and colleagues reported, that at least 80% of ESC guideline committee authors, except for the PD guidelines, had a relevant financial conflict of interest (13). Analysis of conflicts of interest among authors and researchers of European clinical). They propose that physicians involved in the writing of clinical guidelines should be free of financial conflicts of interest “to maintain scientific integrity and independence in the clinical guidelines” (13). However, at present, this approach would mean that clinical experts with conflicts of interest, who possess substantial professional experience and expertise, would be unable to contribute that experience to clinical recommendations (12). To address these issues, mitigation strategies have been developed. Organizations like the World Health Organization and Australia’s National Health and Medical Research Council have implemented policies on disclosure and management of financial conflicts of interest for clinical practice guideline members. In addition, some countries track physicians’ financial payment by industry in public databases rather than relying soley on self-report (14). In our survey respondents were given the option of a free-text response in addition to selecting from predefined answer choices. Although conflicts of interest are cited in the literature as a barrier to implementation as described above, they were not mentioned in our survey as an obstacle to guideline implementation and therefore appear to be of minor relevance for the population we surveyed.

Another obstacle for implementation was, that recommendations of different professional associations to one clinical topic are not always consistent. The discrepancy might be explained by different update dates and thus a more or less current scientific data basis for the recommendation, but in the case of conflicting data, it may also be due to heterogeneous recommendations from different professional associations. One example of this is the current National Care Guideline for Chronic Coronary Heart Disease [Nationale VersorgungsLeitlinie (NVL)-KHK], in which three professional associations involved in its development did not agree with the NVL-KHK in some areas based on scientific evidence and current recommendations from European guidelines and suggested that the guidelines be revised (15). Cases like this do not contribute to confidence in the recommendations. Possible solutions could include the development of indication-specific guidelines in collaboration with relevant professional societies, as well as the development of clear, practice-oriented approaches for dealing with substantive discrepancies.

We observed differences between the subgroups surveyed in terms of how they obtain information: employees in group practices, specialists working as general practitioners, and members of a medical association are more likely to regularly obtain information about current guidelines than doctors in solo practices, doctors who do not belong to a medical association, and general practitioners. This could be due to a stronger focus on information about guideline updates actively provided by medical associations.

Participants in this survey referred as common sources of information certified medical training courses and medical journals, followed by publications from medical associations, information from pharmaceutical sales representatives, participation in scientific conferences, and publications from associations of statutory health insurance physicians. More than half of those surveyed cited short summaries of key content, pocket guidelines from professional associations, discussions in quality circles, brief information in the German Medical Journal “Deutsches Ärzteblatt” which is free assessable for all German physicians, and commentary by experts as supportive measures for implementing clinical guideline recommendations. In a study on the process quality of disseminating information materials on clinical guidelines conducted by the Institute for Quality and Efficiency in Health Care (IQWiG), the researchers investigated which support measures led to an improvement in the dissemination of guideline recommendations. Here, training courses on systematic reviews and training courses conducted by external experts or local opinion leaders were found to improve guideline implementation (16).

We did not observe any preference for analog or digital media when gathering information. Back in 2003, Jeannot and colleagues investigated the potential of the internet to support the implementation of clinical practice guidelines. Two guidelines were developed using a standardized panel method and made available on the web. One concerned the indications for back surgery, the other the indications for upper and lower endoscopy of the digestive tract. A questionnaire was used to record the usage behavior of 20 physicians. In 2003, one limitation was that less than half of the participating physicians had direct access to the internet in the examination room. However, three-quarters of the participants stated that usage was easy and that the average time spent was 3.4 min, or 12% of the time spent with patients. One reason cited for not using the internet was a fear that it would impair the relationship between doctors and patients (17). It is to be expected that digital offerings will also be used to disseminate clinical guidelines in the future. One advantage of this medium could be that, for example, a push function could be used to provide timely notification of updates. A concrete approach has been adopted by the National Health Service [NHS; (18)] in England through the development of a guide that identifies best practices, based on findings from the scientific literature, to support digital and clinical leaders at the local and regional level in the implementation of clinical guidelines. In addition to defining specific success factors, the guide highlights tools that already support the implementation of guideline recommendations today. These include, for example, specific alerts for sepsis within the electronic health record, clinical decision support systems in paediatric intensive care, and digital applications that support cost-effective and safe prescribing (NHS). It is expected that these and similar tools will increasingly support the translation of clinical guidelines into everyday practice in the future.

The Association of Scientific Medical Societies [AWMF; (19)] has established a process that describes the creation and distribution of clinical guideline recommendations in Germany (20), whereby distribution consists of the passive provision of guidelines on the AWMF website, similar to the National Care Guidelines (NVL) program. A reorientation may be imminent here. In the future, editorial and operational coordination will be taken over by the Central Institute for Statutory Health Insurance Medical Care (Zi). The NVL will be published by the Institute for Medical Knowledge Management (IMWi) of the Association of Scientific Medical Societies (AWMF) and the scientific medical societies in the AWMF, following the dissolution of the Medical Center for Quality in Medicine (ÄZQ), which previously coordinated the program.

Since an annual update of the e standardized implementation of clinical guidelines in healthcare. These include standardized and regular monitoring of the level of evidence of clinical guidelines, implementation processes that can be integrated into clinical workflows, and standard review by individual clinicians or healthcare providers should focus on practicality, consideration of patient needs, and the creation of a de-implementation plan to ensure that previously accepted practices can be replaced if current recommendations are found to be ineffective or even harmful (21).

Given the high importance of medical journals in information gathering in our survey, the use of association organs, which reach almost all physicians, could be a good way to communicate updated guideline recommendations.

Our study has limitations. The population surveyed was not selected according to representative criteria. In addition, among all physicians who could potentially be invited, fewer than 10% opened the link to the questionnaire and about half of these completed it. This may have introduced bias. However, the age structure, gender distribution, and proportion of group practices roughly correspond to the current average in German field based practices (22). In our view, the results therefore still allow conclusions to be drawn about the use of guideline recommendations in everyday practice.

The following limitation should also be noted: recruitment was conducted via two different pathways, and during personal contact an information flyer signed by a professional society was used. This may have created social pressure on those approached, which could have influenced their responses. However, since fewer than 8% of participants responded via this route, we consider any potential impact on the overall results to be negligible.

Another limitation is that none of the members of the research team for this paper is a general practitioner. However, the authors, who are members of the scientific societies and were involved in developing the manuscript concept, are familiar with the workflows in referring practices through close contacts with these practices as well as through their activities within the professional societies, whose members largely include physicians in general practices. In addition, there is close exchange between the participating professional societies and physicians in general practices with the aim of integrating the implementation into everyday practice while taking existing workflows into account. Accordingly, the workflows of daily medical practice, as well as the requirements and possibilities with regard to continuing medical education were also considered in the interpretation of the data.

In addition, the questionnaire referred to a limited area of therapy only. However, since cardiovascular diseases account for a large proportion of illnesses in medical practice (23), this focus is certainly relevant for a large patient group who can benefit from these recommendations in their family doctor’s practice.

A further limitation is that some questions used a rating scale with the response options never, rarely, often/frequently, or always. When answering these questions, no specific frequencies based on objective measurement methods were recorded. Consequently, the responses represent a subjective assessment. This should be taken into account when interpreting the results.

As stated in the disclosures, the study was financially supported by APONTIS PHARMA. One of the authors is an employee of the company. This should be taken into account when evaluating the discussion of the results.

Clinical decision-making can be understood as the interface between evidence-based medicine and the demands as well as the constraints imposed by the health care system, the individuality of clinicians, and, above all, the physician–patient relationship. Within this framework, clinical guidelines are not the sole factor, but they are an important one in translating scientific evidence into patient-oriented treatment. In addition to the continuous improvement of the guideline development process, greater efforts should be made to identify barriers to and facilitators of their implementation in routine practice. It would therefore be desirable to examine our observations in a larger, representative population in order to derive concrete recommendations.

## Conclusion

Clinical guideline recommendations are accepted in everyday practice in Germany. However, scope, lack of practical relevance, time required, discrepancies between recommendations, and differences in validity represent hurdles for implementation. Summaries of key recommendations, pocket guidelines, and regular publications in journals could help to increase efficiency and application in practice. In addition, it would be useful to establish a standardized implementation process for patient-relevant recommendations in everyday practice, ensuring care in accordance with the best medical evidence.

## Data Availability

The raw data supporting the conclusions of this article will be made available by the authors, without undue reservation.
