# Supplementary material for: Evaluation of clinical guideline implementations for cardiovascular diseases by German general practitioners—Results of a web-based survey
Source: Front Med (Lausanne). 2026 Jul 3;13:1852365. doi: 10.3389/fmed.2026.1852365 (PMC13377685; doi:10.3389/fmed.2026.1852365)
Supplement: Supplementary file 1 [file Data_Sheet_1.pdf]

General information about you and your practice
(Multiple answers possible)

General practitioner	☐
Specialist working as a family doctor	☐
Practical doctor	☐
Group practice	☐ yes ☐ no
First three digits of the practice's postal code	I__I__I__I

Gender ☐ female ☐ male ☐ diverse

Age
☐ up to 30
☐ 31-40
☐ 41-50
☐ 51-60
☐ older than 60

Are you a member of the following professional associations?
(Multiple answers possible; the exact name of each professional association is provided when you hover over it with the mouse.)

Member of DHL	☐ yes	☐ no
Member of DGPR	☐ yes	☐ no
Member of DGFF	☐ yes	☐ no
Member of DGK	☐ yes	☐ no
Member of DEGAM	☐ yes	☐ no

Member of other professional association _ ____

ALL:
How often do you implement guideline recommendations for cardiovascular diseases in your daily practice?

Never	Rarely	Frequently	Always
☐	☐	☐	☐

FILTER: if guideline use ~= always
What prevents you from implementing guideline recommendations?
(display randomly)

	Yes	Somewhat yes	Somewhat no	No
Guidelines too comprehensive	☐	☐	☐	☐
Recommendations deviate from other guidelines	☐	☐	☐	☐
Updates too frequent	☐	☐	☐	☐
Uncertainty as to which guideline is valid	☐	☐	☐	☐
Takes too much time	☐	☐	☐	☐
Difficult or cumbersome to find	☐	☐	☐	☐
Guideline recommendations are too impractical	☐	☐	☐	☐
Other reasons				

FILTER: If guideline use ~= never
What motivates you to implement the guideline recommendations?
(display randomly)

	Yes	Somewhat yes	Somewhat no	No
Degree of recommendation in guidelines	☐	☐	☐	☐
Recommending professional association	☐	☐	☐	☐
Discussion in certified medical	☐	☐	☐	☐
continuing education events	☐	☐	☐	☐
Other________________________________				

FILTER: If guideline use ~= never
Which guidelines do you follow in your daily practice?

☐ National care guidelines
☐ Guidelines from a national professional association
☐ Guidelines from an international professional association
☐ Guidelines from the World Health Organization (WHO)

FILTER: Display of guidelines observed; question is only asked if more than one guideline is used.
Please rate on a scale of 0-10 how important the guidelines you use are to you in your daily practice. 0 stands for not important, 10 for very important.
OR
Please use the slider to rate how important the guidelines you use are to you in your daily practice.
National care guideline
Guideline from a national professional association
Guideline from an international professional association
Guideline from the World Health Organization (WHO)

ALL:
What [if guideline use = never: could/otherwise: can] support you in implementing guideline recommendations in your practice?
(Multiple answers possible)

	yes	somewhat yes	somewhat no	no
Brief summary of key recommendations	☐	☐	☐	☐
Pocket guidelines from professional associations	☐	☐	☐	☐
Discussion, e.g., in medical quality circles	☐	☐	☐	☐
Brief information on key aspects in the German Medical Journal	☐	☐	☐	☐
Comments on key content by experts	☐	☐	☐	☐
Other___________________________				

FILTER: If yes/somewhat yes:
In what form would commentary on essential content by experts be helpful to you?
☐ As a digital contribution, e.g., as a podcast/video
☐ As a print version

ALL:
Do you regularly check for new/
current guidelines on cardiovascular diseases? ☐ yes ☐ no

FILTER: If regular information = yes

	never	rarely	often	always
I obtain information about guidelines	☐	☐	☐	☐
Through publications by professional associations	☐	☐	☐	☐
Through participation in scientific conferences	☐	☐	☐	☐
Through publications by the KVs	☐	☐	☐	☐
Via professional journals	☐	☐	☐	☐
Via participation in certified medical training Courses	☐	☐	☐	☐
Via pharmaceutical representatives	☐	☐	☐	☐
Via medical scientific liaison (MSL)	☐	☐	☐	☐
Other________________________________				
