# Supplementary material for: Evaluation of clinical guideline implementations for cardiovascular diseases by German general practitioners—Results of a web-based survey
Source: Front Med (Lausanne). 2026 Jul 3;13:1852365. doi: 10.3389/fmed.2026.1852365 (PMC13377685; doi:10.3389/fmed.2026.1852365)
Supplement: Supplementary file 2 [file Supplementary_file_2.pdf]

Checklist for Reporting Of Survey Studies (CROSS)

Section/topic	Item	Item description
**Title and abstract**		
Title and abstract		**State the word "survey" along with a commonly used term in title or abstract to introduce the study's design.**<br>*Evaluation of clinical guideline implementations for cardiovascular diseases by German general practitioners – results of a web-based survey*<br>**Provide an informative summary in the abstract, covering background, objectives, methods, findings/results, interpretation/discussion, and conclusions.**<br>*Clinical guidelines are intended to improve the quality of medical care. The aim of this survey was to record the concrete implementation of guidelines in practice and criteria for their implementation in Germany. Using a web-based questionnaire developed with medical scientific societies and uzbonn, general practitioners were able to participate anonymously in the survey between July 1, 2024, and October 31, 2024. Data were statistically evaluated using descriptive methods. 437 physicians participated in the survey. 74.1% frequently implement guideline recommendations, 13.3% always, 11.2% rarely, and 1.4% never. 67.3% consider the guidelines to be too extensive. 71.6% regularly inform themselves about guideline updates. Motivators include a high recommendation rate (86.4%), recommending scientific society (78.7%), and discussion in medical training events (85.6%). The highest attention is given to National Care Guidelines (Nationale VersorgungsLeitlinie, NVL) 71.2% and guidelines of national scientific societies (65.4%). Barriers include the extent (67.3%), lack of practical relevance (51.5%), time required (47.2%), differing recommendations between various guidelines (46%), and uncertainty about validity (45.9%). Supportive measures would be a summary of essential content (98%), pocket guidelines from scientific societies (84.2%), discussions in quality circles (76.4%), and brief information in German medical journals (73.2%).*<br>*Clinical guideline recommendations are highly accepted in everyday practice, especially the German NVL. The extent, lack of practical relevance, time required, discrepancies in recommendations, and differences in validity are significant barriers. Summaries of essential recommendations, pocket guidelines, and regular publications in medical journals could help increase application of clinical guidelines in practice.*
**Introduction**		
Background		***Provide a background about the rationale of study, what has been previously done, and why this survey is needed.***<br>*Clinical guidelines are systematically developed decision-making aids for physicians with the aim of improving the quality of medical care. The recommendations are to be implemented in everyday practice through knowledge transfer. Taking clinical guidelines into account in everyday practice*

	can have a significant impact on patient health. Initial studies on the implementation of treatment recommendations in secondary prevention of cardiovascular disease in German medical practices indicate that non-compliance results in higher morbidity and mortality than consistent implementation. However, information on how often clinical guidelines are implemented in daily practice and which factors hinder or increase implementation are still missing.
Purpose/aim	**Identify specific purposes, aims, goals, or objectives of the study.**<br>*The aim of our study was to determine 1. how often clinical guidelines are implemented in daily practice, 2. which factors hinder implementation, and 3. which measures could increase implementation.*
**Methods**	
Study design	**Specify the study design in the methods section with a commonly used term (e.g., cross-sectional or longitudinal).**<br>*This study used a cross-sectional, self-administered web-based survey design.*
Data collection methods	**Describe the questionnaire (e.g., number of sections, number of questions, number and names of instruments used).**<br>*The questionnaire was divided into two sections: general questions about the practice (4 questions) and question-specific questions (10 questions).*<br>**Describe all questionnaire instruments that were used in the survey to measure particular concepts. Report target population, reported validity and reliability information, scoring/classification procedure, and reference links (if any).**<br>*The questionnaire consisted with closed-ended items, mostly using four-point agreement or frequency scales.*<br>**Provide information on pretesting of the questionnaire, if performed (in the article or in an online supplement). Report the method of pretesting, number of times questionnaire was pre-tested, number and demographics of participants used for pretesting, and the level of similarity of demographics between pre-testing participants and sample population.**<br>*Prior to the launch all surveys underwent both automatic and manual testing. By generating random test cases, the survey system allowed precise verification of the filter guidance. In addition, the questionnaires were thoroughly checked by a team of qualified testers to ensure that the programming fully corresponded to the template. A secure connection (https) was used for the online test questionnaires to adequately protect the data entered.*<br>**Questionnaire if possible, should be fully provided (in the article, or as appendices or as an online supplement).**<br>*Questionnaire will be fully provided in the supplementary material.*

Checklist for Reporting Of Survey Studies (CROSS)

	**Describe the study population (i.e., background, locations, eligibility criteria for participant inclusion in survey, exclusion criteria).**<br>*Study population were German general practitioners.*<br>**Describe the sampling techniques used (e.g., single stage or multistage sampling, simple random sampling, stratified sampling, cluster sampling, convenience sampling). Specify the locations of sample participants whenever clustered sampling was applied**.<br>*German general practitioners were asked by pharmaceutical representatives of APONTIS PHARMA Deutschland GmbH & Co. KG to give their consent to be contacted by mail regarding medical-scientific information and/or medical-scientific surveys. A total of 12,000 who provided written consent to such contact were invited by e-mail and 1,000 via printed flyers; the invitation described the study content, emphasized independent data analysis, and disclosed funding by APONTIS PHARMA Deutschland GmbH & Co. KG.*
Sample characteristics	**Describe how representative the sample is of the study population (or target population if possible), particularly for population-based surveys.**<br>*The population surveyed was not selected according to representative criteria. In addition, among all physicians who could potentially be invited, fewer than 10% opened the link to the questionnaire and about half of these completed it. This may have introduced bias. However, the age structure, gender distribution, and proportion of group practices roughly correspond to the current average in German field based practices (National Association of Statutory Health Insurance Physicians, 2025).*<br>**Provide information of survey's time frame, such as periods of recruitment, exposure, and follow-up days.**<br>*German general practitioners were able to participate anonymously in the survey between July 1, 2024, and October 31, 2024.*<br>**Provide information on the entry process:**<br>*Access to the questionnaire was provided via an SSL-encrypted connection with all pages displayed as standard web forms.*
Study preparation	**Describe any preparation process before conducting the survey (e.g., interviewers' training process, advertising the survey).**<br>*The questionnaire was developed in collaboration with German medical societies (German Hypertension League (Deutsche Hochdruckliga e.V. DHL®), German Society for Prevention and Rehabilitation of Cardiovascular Diseases (Deutsche Gesellschaft für Prävention und Rehabilitation von Herz-Kreislauferkrankungen e.V.), German Society for the Control of Lipid Metabolic Disorders and their Consequences (Deutsche Gesellschaft zur Bekämpfung von Fettstoffwechselstörungen und ihren Folgeerkrankungen e.V.), and uzbonn using an iterative process involving an expert advisory board and a workshop, with a focus on valid operationalization of the research questions.*

Ethical considerations

Provide information on ethical approval for the survey if obtained, including informed consent, institutional review board [IRB] approval, Helsinki declaration, and good clinical practice [GCP] declaration (as appropriate).

According to national regulations for anonymous survey research among physicians, formal approval by an ethics committee and written informed consent were not required.

Provide information about survey anonymity and confidentiality and describe what mechanisms were used to protect unauthorized access.

Participation in the survey was entirely voluntary. Before starting the questionnaire, participants were informed about the purpose of the study, the anonymous and aggregated analysis of the data, and the funding source; proceeding to the first survey page was considered as providing informed consent. All current requirements in guidelines for observational/claims-based data studies in Germany, legal requirements and in particular requirements regarding data protection and the protection of individuals were followed in this study.

Statistical analysis

Describe statistical methods and analytical approach. Report the statistical software that was used for data analysis.

All analyses were performed using IBM SPSS Statistics.

Results

Respondent characteristics

Report numbers of individuals at each stage of the study. Consider using a flow diagram, if possible.

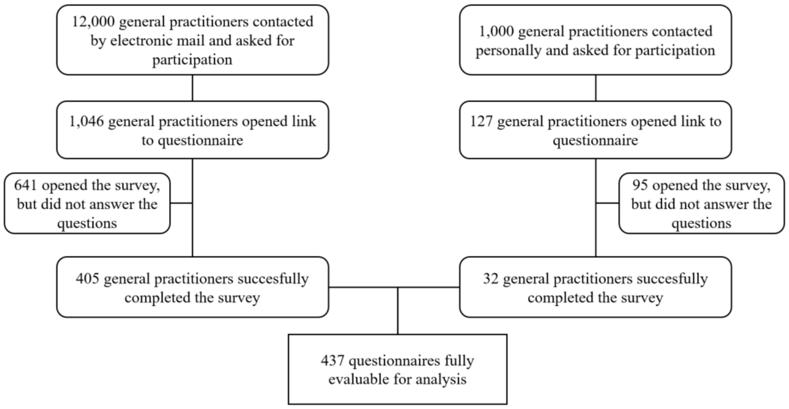

Provide reasons for non-participation at each stage, if possible.

See CONSORT diagram above.

Report response rate, present the definition of response rate or the formula used to calculate response rate.

Of 13,000 physicians invited, 1,046 (8.0%) accessed the survey link and 437 (3.4% of all invitees; 41.8% of those who opened the link) completed the questionnaire and were

	included in the analysis.
Descriptive results	**Provide characteristics of study participants, as well as information on potential confounders and assessed outcomes.**<br>*57% of participants were general practitioners, 43% were specialists working as family doctors (14 respondents did not provide information). 45% of participants stated that they worked in a group practice (2 respondents did not provide information). 53% of participants were male. 38.7% were between 51 and 60 years old, 27.5% were older than 60, 24.7% were between 41 and 50, 8.2% were between 31 and 40, and 0.5% were 30 or younger (0.5% did not specify their age).*<br>*23% of respondents were members of the German Society for General Medicine and Family Medicine (Deutsche Gesellschaft für Allgemeinmedizin und Familienmedizin, DEGAM), 8% were members of the German Society for Cardiology (Deutsche Gesellschaft für Kardiologie , DGK), 8% were members of the German Hypertension League (DHL), 1% were members of the German Society for the Prevention of Lipid Metabolism Disorders and their Consequences (DGFF), and 1% were members of the German Society for the Prevention and Rehabilitation of Cardiovascular Diseases (DGPR). 35% were members of another professional association, and 41.4% of respondents did not belong to any professional association (multiple answers possible).*
**Discussion**	
Limitations	**Discuss the limitations of the study, considering sources of potential biases and imprecisions, such as non-representativeness of sample, study design, important uncontrolled confounders**.<br>*The population surveyed was not selected according to representative criteria. In addition, among all physicians who could potentially be invited, fewer than 10% opened the link to the questionnaire and about half of these completed it. This may have introduced bias. However, the age structure, gender distribution, and proportion of group practices roughly correspond to the current average in German field based practices (National Association of Statutory Health Insurance Physicians, 2025). In our view, the results therefore still allow conclusions to be drawn about the use of guideline recommendations in everyday practice.*<br>*The following limitation should also be noted: recruitment was conducted via two different pathways, and during personal contact an information flyer signed by a professional society was used. This may have created social pressure on those approached, which could have influenced their responses. However, since fewer than 8% of participants responded via this route, we consider any potential impact on the overall results to be negligible.*<br>*Another limitation is that none of the members of the research team for this paper is a general practitioner. However, the authors, who are members of the scientific societies and were involved in developing the manuscript concept, are familiar with the workflows in referring practices through close contacts with these practices as well as through their activities within the professional societies, whose members largely include physicians in general practices. In addition, there is close exchange between the participating professional societies and physicians in general practices with the aim of integrating the implementation into everyday practice while taking existing workflows*

	into account. Accordingly, the workflows of daily medical practice, as well as the requirements and possibilities with regard to continuing medical education were also considered in the interpretation of the data.<br>*In addition, the questionnaire referred to a limited area of therapy only. However, since cardiovascular diseases account for a large proportion of illnesses in medical practice (Deutsches Ärzteblatt, 2024), this focus is certainly relevant for a large patient group who can benefit from these recommendations in their family doctor's practice.*<br>*A further limitation is that some questions used a rating scale with the response options never, rarely, often/frequently, or always. When answering these questions, no specific frequencies based on objective measurement methods were recorded. Consequently, the responses represent a subjective assessment. This should be taken into account when interpreting the results.*<br>*As stated in the disclosures, the study was financially supported by APONTIS PHARMA. One of the authors is an employee of the company. This should be taken into account when evaluating the discussion of the results.*
Interpretations	**Give a cautious overall interpretation of results, based on potential biases and imprecisions and suggest areas for future research**.
Generalizability	*Clinical guideline recommendations are accepted in everyday practice in Germany. However, scope, lack of practical relevance, time required, discrepancies between recommendations, and differences in validity represent hurdles for implementation. Summaries of key recommendations, pocket guidelines, and regular publications in journals could help to increase efficiency and application in practice. In addition, it would be useful to establish a standardized implementation process for patient-relevant recommendations in everyday practice, ensuring care in accordance with the best medical evidence.*
**Other sections**	
Role of funding source	**State whether any funding organization has had any roles in the survey's design, implementation, and analysis.**<br>*This study was financed by Apontis Pharma Deutschland GmbH & Co. KG, Monheim, Germany. No influence was exerted on the study design, data analysis and interpretation.*
Conflict of interest	**Declare any potential conflict of interest**.<br>*Olaf Randerath was employed by APONTIS PHARMA Deutschland GmbH & Co. KG. The remaining authors declare that the research was conducted in the absence of any commercial or financial relationships that could be construed as a potential conflict of interest.*
Acknowledgements	**Provide names of organizations/persons that are acknowledged along with their contribution to the research.**<br>*Van der Giet M, Schwaab B, Weingärtner O: Conceptualization – Methodology – Investigation - Writing original draft; Mayerböck A: Methology – Investigation - Data curation – Resources – Software – Validation – Vizualisation - Writing – review &*

editing; Klein K: Investigation – Formal analysis - Data curation – Software – Validation – Vizualisation - Writing – review & editing; Randerath O: Project administration - Writing original draft
